# Clinical outcomes of arthroscopic surgery for external snapping hip

**DOI:** 10.1186/s13018-017-0584-1

**Published:** 2017-06-02

**Authors:** Amrit Shrestha, Peng Wu, Heng’an Ge, Biao Cheng

**Affiliations:** Department of Orthopedics, Shanghai Tenth People’s Hospital, Tongji University School of Medicine, No. 301 Yanchang Middle Road, Jing’an District, Shanghai, 200072 China

**Keywords:** External snapping hip (ESH), Arthroscopy, Classification, Outcome

## Abstract

**Background:**

Studies have reported on the arthroscopic technique for release of external snapping hip syndrome. However, no study with large sample size has been reported for arthroscopic surgery.

**Methods:**

Patients with 229 bilateral and 19 unilateral external snapping hips were treated from January 2012 to June 2013. After locating the contracture position, arthroscopic surgery was performed accordingly. Preoperative and postoperative angles were compared.

**Results:**

Comparing range of motion, all patients obtained higher adduction and flexion angles. At postoperative follow-up of 24 months, the adduction angle was improved from −14.4 ± 5.14 to 35.7 ± 4.21 for type I, from −31.2 ± 5.22 to 31.7 ± 2.84 for type II, from −49.0 ± 3.47 to 21.6 ± 3.43 for type III, and from −64.5 ± 4.65 to 18.3 ± 3.10 for type IV (*P* < 0.001). Similarly, the flexion angle was also significantly improved for all the four types (*P* < 0.001). Excellent ratio and satisfaction rate were good in types I and II. All the clinical features were cured after arthroscopic surgery.

**Conclusions:**

Arthroscopic surgery could be an effective procedure for external snapping hip, due to less operating time, small scar, fast postoperative recovery, and complete contracture release.

## Background

Snapping hip syndrome (SHS) is characterized with an audible or palpable snap when flexing or extending the hip and sometimes can be associated with pain [1, 2]. Based on the causes, it can be divided into two types, intra-articular or extra-articular [3]. Intra-articular is mainly referred to the lesion in the joint itself, including synovial chondromatosis, labral tears, and fracture fragments or loose bodies. Extra-articular is the most common form of snapping hip that affects structures including the proximal hamstring tendon, the iliotibial band (ITB), the fascia lata, or the gluteus maximus (GM). Extra-articular is further classified into two types, internal and external snapping hip (ESH). ESH usually occurs with flexion and extension of the hip during exercise when the thick taut posterior border of the ITB moves over the great trochanter (GT) [4, 5].

**Fig. 1:**
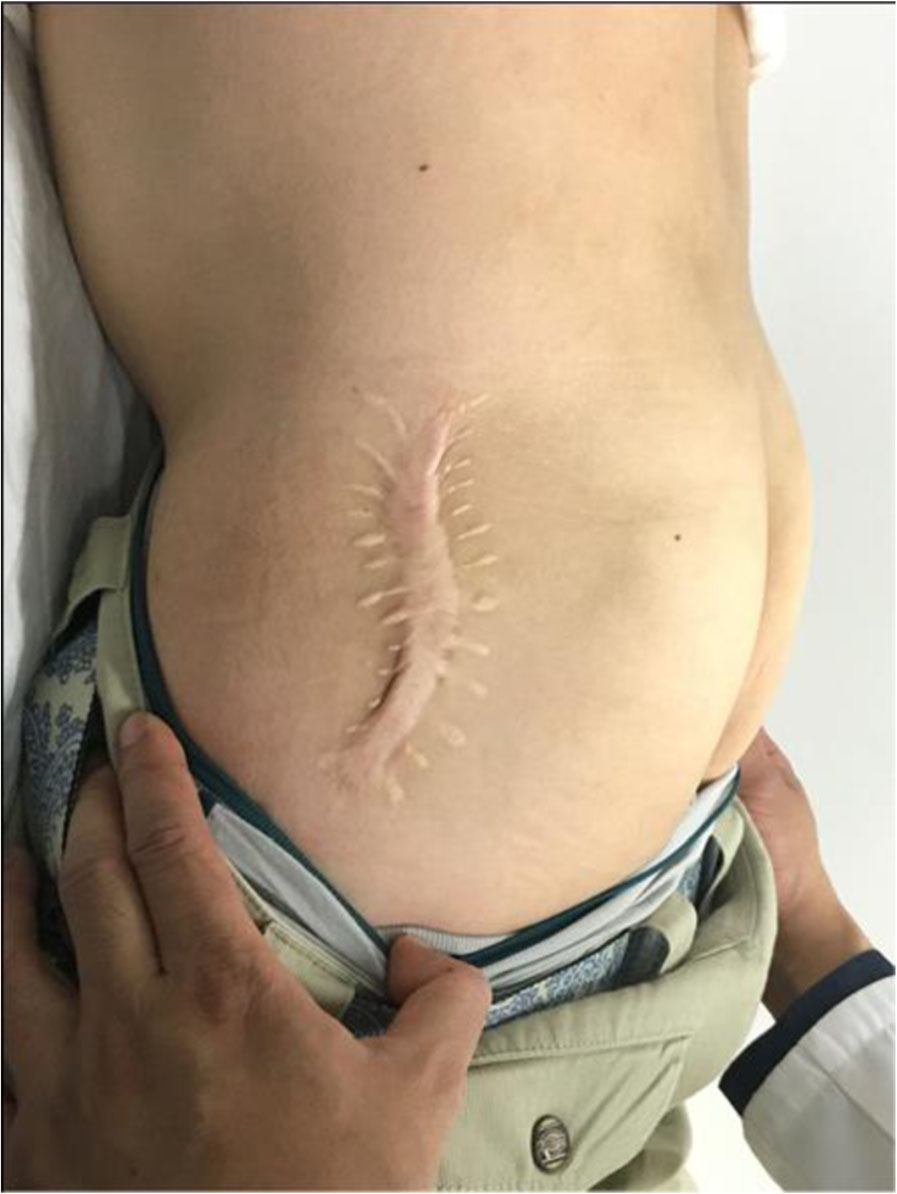
Patient with hypertropic scar after open surgery. This patient came to our hospital for arthroscopic surgery after failure of open surgery. The huge scarring in the incision can also been seen

Commonly, the first step of management of ESH is conservative. This consists of rest, avoiding movements that provoke snapping, stretching exercise, non-steroidal anti-inflammatory drugs (NSAID), and injections of steroid into the trochantric bursa [6, 7]. Once conservative treatment is useless for the contracture, surgical release is necessary. Various open procedures of ITB release have been described, such as Z-plasty, N-plasty with trochanteric bursectomy, ellipsoid resection of the tract over the trochanter, cruciate incision with sutured flaps to the tract, and resection of the posterior half of the tract at the GM insertion [6, 8, 9]. Each of these procedures has had varying degrees of success in the contracture release. Besides, extensive surgical trauma, hematoma formation, wound complication, and slow postoperative recoveries are the drawbacks of these traditional open surgeries (Fig. 1) [10].

Recently, arthroscopic technique to release the ITB in patients with ESH was introduced with excellent contracture release and fast recovery [3, 11]. In 2006, Ilizaliturri et al. [12] was the first to report arthroscopy surgery for ESH. Since then, various methods of arthroscopy have been reported to treat ESH, and it depends on surgeon’s preference and the pathology being treated. With reviewing the published literature online, we found there is no article of arthroscopy to treat ESH in which sample size was more than 100. The exact outcomes for the treatment of ESH under arthroscopy remain unclear. Therefore, we conducted this study with large sample size to assess the outcomes for the treatment of ESH patients with different severity according to different types of ESH.

## Methods

This study was approved by the Institutional Review Board of Shanghai Tenth People’s Hospital affiliated to Tongji University. Each subject provided his or her written informed consent.

Between January 2012 and June 2013, a total of 248 patients (99 male and 149 female) with ESH syndrome treated in our hospital by arthroscopic surgery were included in this study, based on the inclusion criteria. Patients mainly presented themselves with complaints of snapping, clicking or popping sound heard when squatting from the standing position or during jugging at the lateral upper thigh over the area of GT, sometimes accompanied by pain. Furthermore, all patients experienced this disorder from repeated injection on hip during their childhood. Inclusion criteria were set as follows: (1) all patients diagnosed with ESH by history and physical examination, (2) all patients aged between 16 and 40 years old, (3) all patients received the contracture release under arthroscopy, (4) a minimum of 2-year follow-up, and (5) available data for hip assessment. Exclusion criteria were (1) the presence of intra-articular disease, (2) the presence of bony deformities, and (3) the patients with chronic disease or infections.

All patients experienced failed conservative treatment which is used for each patient for a period of at least 3 months before surgery. Passive and active stretching was applied to increase muscle length. Eccentric control is trained to modify neuromuscular control to allow muscle lengthening. Modification of movement patterns (gains) and consistent stretching can be helpful to prevent recurrence. Rest, icing, and anti-inflammatories were advocated to avoid inflammation of tissues.

**Fig. 2:**
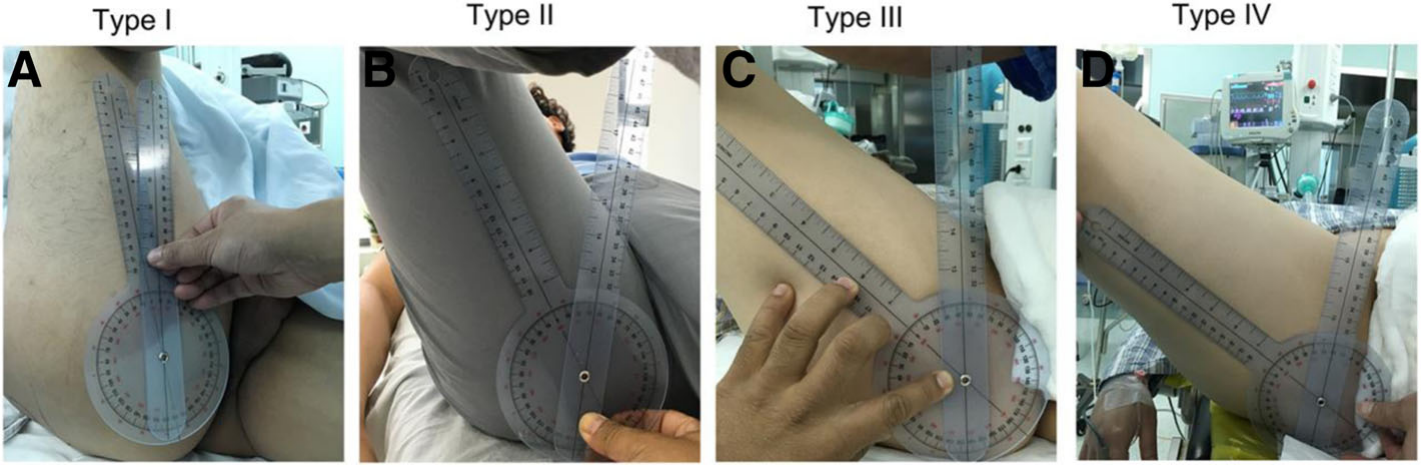
Range of motions of different types of ESH. **a** Adduction angle is −10° with hip and knee joint in 90° for type I patient. **b** Adduction angle is −35° with hip and knee joint in 90° for type II patient. **c** Adduction angle is −45° with hip and knee joint in 90° for type III patient. **d** Adduction angle is −60° with hip and knee joint in 90° for type IV patient

**Fig. 3:**
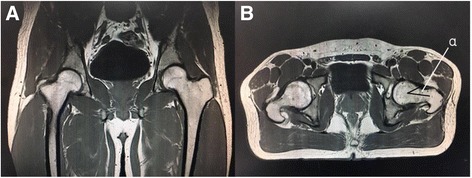
Differential diagnosis of intra-articular pathologies in MRI images. **a** Normal hip in coronal section. **b** Normal angle *α* showed in transverse section

Before surgery, we performed the preoperative evaluation including physical and radiographic examinations. Preoperative range of motion of hip was measured (Fig. 2). A hip MRI was taken to identify if any bony abnormalities, calcifications, avulsion of GT, loss of joint space, pincer lesions, acetabular dysplasia, or other pathologies were existed (Fig. 3). Based on the angle of adduction with flexion of hip in 90°, patients were graded into four types (Table 1).

**Table 1:** Classification for the location of contraction of external snapping hip

Type	Angle of hip
I	Hip adduction −5° to −20° with hip and knee joint flexion in 90°
II	Hip adduction −20° to −40° with hip and knee joint flexion in 90°
III	Hip adduction −40° to −60° with hip and knee joint flexion in 90°
IV	Hip adduction >−60° with hip and knee joint flexion in 90°

**Fig. 4:**
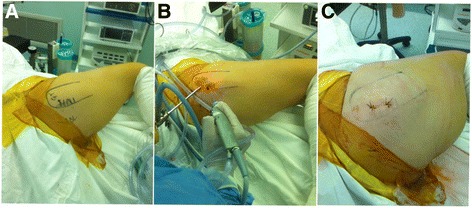
Operative position and portals. **a** Clinical photograph showing important landmarks: *GT* (greater trochanter), *SN* (sciatic nerve), and position of the two portals. Proximal and distal portals are marked 3–4 cm apart on GT. **b** Standard 30° scope is inserted through the proximal portal in 30° angle. Shaver and radiofrequency device is inserted through the distal portal. **c** After the surgery was completed, the portals are closed

**Fig. 5:**
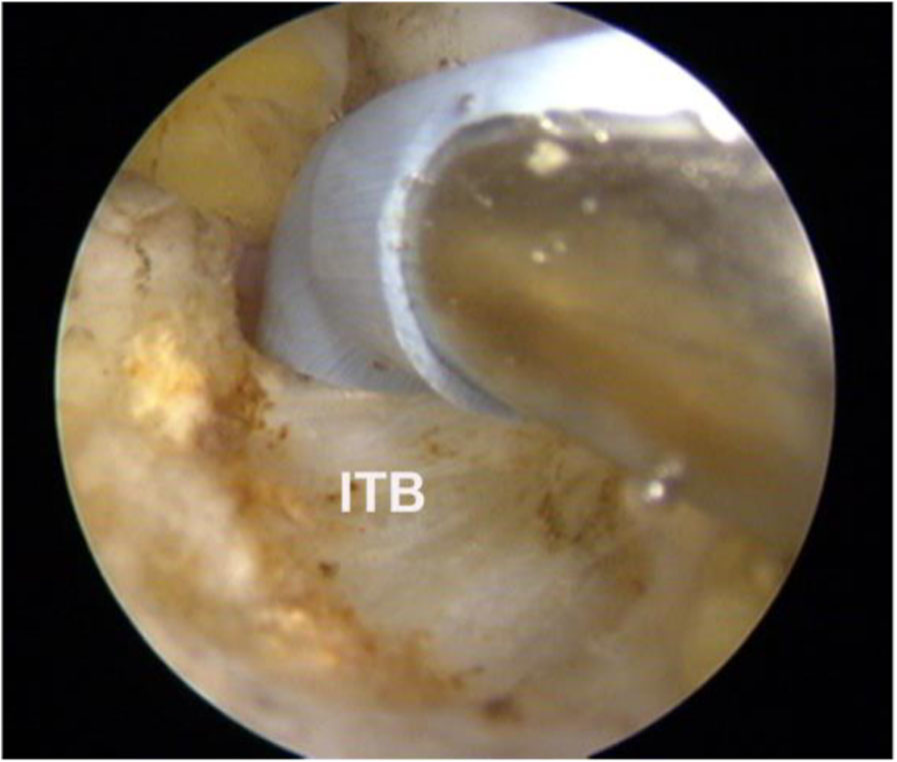
Intraoperative view. The picture suggests the intraoperative view under arthroscopy showing radiofrequency device cutting the iliotibial band (*ITB*)

All the arthroscopy operations were performed under general anesthesia. Patient was placed in lateral decubitus position on a standard operating table. The hip being flexed, adducted, and internally rotated to the maximum possible degree without traction was the position of the operation. Standard sterile draping was done. Two portals were approximately 3–4 cm apart and were marked over the GT. For portals, oblique incision of 3 mm in size was made on the skin and subcutaneous tissue. A standard 30° scope with a diameter of 4 mm was inserted through proximal portal at a 30° angle (Fig. 4). For good vision of the peritrochanteric space, 40 ml of normal saline was pumped at low pressure during surgery. Through inserting the distal portal saver, the fat and fibrous tissues were cleaned in the operating space. Shaver was removed and radiofrequency device was inserted through the same distal portal. ITB (Fig. 5) was initially cut partially from both anterior and posterior sides. Only after final exploration was done, the remaining part of ITB was cut completely whereas contractures of GM and tensor fascia lata (TFL) bands were cut completely at once. After the contractures were removed, the leg was slowly moved to through a full range of motion (ROM) of the hip to confirm no clicking sounded. The sciatic nerve should be considered to avoid its injury when operating. In presence of any bleeding point, cautery can be used. Once the surgeon was satisfied, fluid was aspirated and the skin was sutured to close the portals. A similar process was done on the other side. The total duration of operation was approximately 15–20 min.

After surgery, non-steroidal anti-inflammatory drugs and ice therapy were used for pain release for just 3 days postoperatively. The patients were encouraged to flex the hip and knee joint and cross the legs. The rehabilitation program was suggested to achieve rapid recovery until 6 months postoperatively. Postoperative rehabilitation was same for each patient. Patients were evaluated preoperatively and at 3, 6, 12, and 24 months postoperatively. At each follow-up, physical examination and questionnaire were performed. If subjects could obtain completely recovered, they were identified as excellent for type I or an adduction angle of hip that increased >30° for type II, 45° for type III, and 60° for type IV.

All statistical analyses were performed using SPSS 19.0 software. All preoperative and postoperative indices were compared by a paired *t* test. For ratio comparison, chi-square was performed. A *P* value <0.05 was considered statistically significant.

## Results

In this study, there were 248 patients who received arthroscopic surgery to treat ESH (Table 2). The mean age of the patient was 26 years old (range 8–38 years old) with an average body mass index (BMI) being 22 kg/m^2^ (range 17.7–29). Median duration of symptom was 10 years (range 1 month–30 years). Among the 248 patients, 76 were diagnosed with type I, 83 with type II, 55 with type III, and 34 with type IV.

**Table 2:** Baseline characteristics of the included patients

Variables	
No. of patients	248
Age, mean (range)	26 (8–38)
Gender (M/F)	99/149 (40%/60%)
Symptoms	
Knee pain	15 (6.0%)
Snapping	248 (100%)
Duration of symptoms, median (range)	10 years (1 month–30 years)
Total days in hospital, days, mean (range)	6 [1–19]
Length of postoperative hospital stay, days, mean (range)	3 [1–9]
BMI, median (range)	22.0 (17.7–29.0)

*M/F*, male/female

**Fig. 6:**
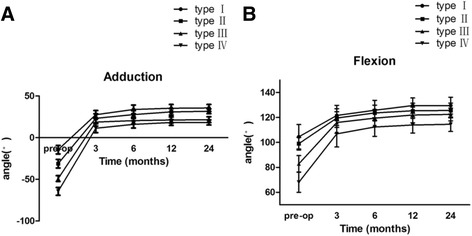
Improvements of range of motion after surgery**. a** Patients have significantly better adduction angle after surgery, particularly in 3-month follow-up. Similar with **a**, **b** also shows significant improvement in the flexion of patients

With regard to ROM, compared with preoperative examination, all patients obtained higher adduction and flexion angles (Table 3). At postoperative follow-up of 24 months, the adduction angle and flexion angle were improved for each type of ESH (Fig. 6). We had also compared ROM between males and females; however, there is no difference between different genders (Table 4).

**Table 3:** Comparison of pre-operation and post-operation in range of motion

	Adduction		*P* value	Flexion		*P* value
	Pre-op	Post-24		Pre-op	Post-24	
Type I	−14.4 ± 5.14	35.7 ± 4.21	<0.001	104.5 ± 9.84	129.5 ± 6.72	<0.001
Type II	−31.2 ± 5.22	31.7 ± 2.84	<0.001	99 ± 5.03	125.6 ± 5.89	<0.001
Type III	−49.0 ± 3.47	21.6 ± 3.43	<0.001	82.9 ± 6.60	122.5 ± 5.12	<0.001
Type IV	−64.5 ± 4.65	18.3 ± 3.10	<0.001	68.1 ± 8.02	114.5 ± 5.75	<0.001

*Pre-op* pre-operation, *post-24* 24 months follow-up postoperatively

**Table 4:** Comparison of range of motion post-operatively according to gender

	Adduction		*P*-value	Flexion		*P*-value
	Male	Female		Male	Female	
Type I	35.2 ± 4.23	35.8 ± 4.20	> 0.05	130.5 ± 7.74	128.9 ± 6.43	> 0.05
Type II	32.3 ± 2.92	31.4 ± 2.76	> 0.05	124.8 ± 5.72	126.7 ± 5.99	> 0.05
Type III	21.9 ± 3.42	21.5 ± 3.49	> 0.05	121.5 ± 5.21	123.6 ± 5.08	> 0.05
Type IV	18.6 ± 3.35	18.2 ± 3.02	> 0.05	113.8 ± 6.02	114.7 ± 5.69	> 0.05

**Fig. 7:**
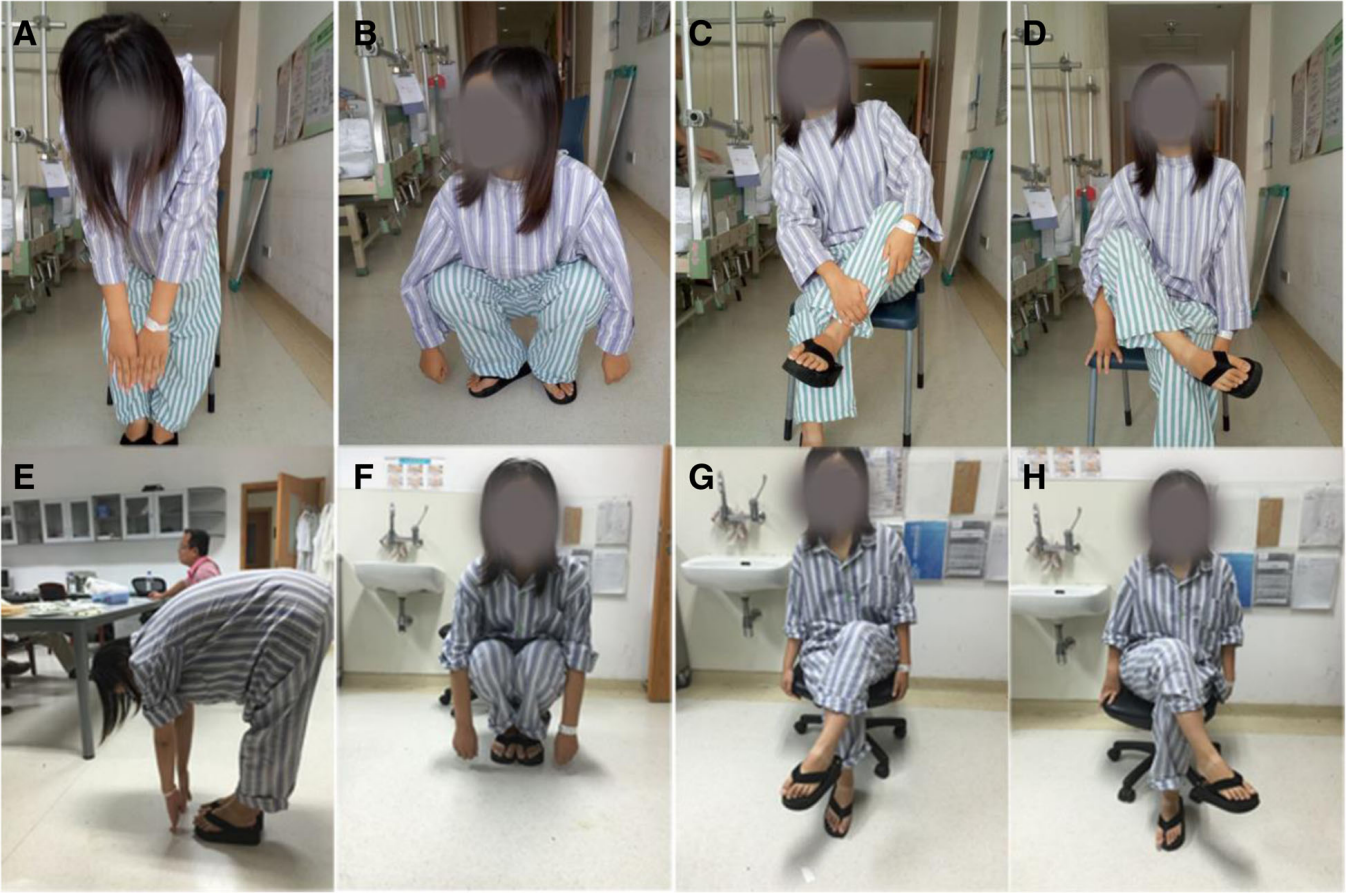
Comparison of range of motion between preoperative and postoperative management**. a** Before surgery, the patient is unable to touch the big toe while flexing the spine with the knee straight. **b** Fixed hip abduction and external rotation are seen during crouching leading to frog leg position. **c**, **d** The patient is unable to sit with her legs crossed. **e** After arthroscopic surgery, the patient is able to touch the big toe while flexing the spine with the knee straight. **f** Frog leg deformity is corrected; the patient can crouch with the both knees together. **g**, **h** The patient is able to sit with her legs crossed

After surgery, no long-term postoperative complications were found in this study, including permanent muscle weakness, neural injury (sciatic nerve), and vascular injury. No infections occurred in the series. Moreover, there was no major swelling, hematomas, and wound dehiscence in these cases. All patients could sit with their legs crossed (Fig. 7). Neither out-toe gait nor Ober’s sign was observed, and there were no recurrent contracture of hip abductors, no snapping, and no residual hip pain or gluteal muscle wasting were seen. There are 15 patients with associated knee pain. After surgery, knee pains of these patients were released.

The outcome of surgery in type I and type II were significantly higher than in type III and IV patients (*P* < 0.05) (Table 5). The excellent ratio in type I [76/76 (100%)] and type II [83/83 (100%)] was higher than in type III [51/55 (92.7%)] and type IV [25/34 (73.5%)]. The satisfaction rate was higher in types II [67/69 (97.1%)] and III [53/55 (96.4%)] than in type I [73/76 (96.1%)]. Although the satisfaction rate of type IV [32/34 (94.1%)] is comparatively lower than in other three types, patient compliance were very good and happy with the results.

## Discussion

In the current study, we included a total of 248 patients to investigate the outcomes of treatment of ESH under arthroscopy. After surgery, we found all patients received the contracture released. Although outcomes in types I and II are significantly higher than those in types III and IV, there is no difference with the patients’ satisfaction in four types.

ESH syndrome was first reported by Valderrama in 1969 [13]. Although multiple factors played roles in the development of ESH, the most common factor was a history of repeated injections in the buttocks, based on the previous reports [14, 15]. ESH is described as hip snapping during moving the hip, mainly due to the thickening of the posterior part of ITB or the anterior border of the GM sliding over the GT [4]. It is commonly seen in athletes like ballet dancers, runners, and soccer players [4]. The pathological changes result in limitation of hip movement with abnormal gait [10, 16, 17]. In some cases, anatomical deformities like oblique pelvis, compensatory scoliosis, and bilateral dislocation of the hip joints are seen [15, 18, 19]. Other impairments of daily activities include being unable to sit with the legs crossed, difficulty in tying shoe laces, and, for some, difficulty in driving. Inflammation of the underlying bursa caused by sliding of the ITB over the GT results in painful snapping to the patient [4, 9].

Commonly, a program of conservative management for systematic ESH is applied first. In case that conservative therapy failed to treat with ESH, a variety of surgical techniques are attempted with variable success. Zoltan et al. [20] performed open procedure with significantly improved or relieved symptoms. Zhao et al. [21] also used an open surgical technique to treat with ESH. After follow-up, they showed that operative management was effective in patients at all levels and suggested that either conservative or operative management should be conducted as early as possible. White et al. [2] reported open procedure for ESH in 16 patients with improvement of the pain and snapping in 14 patients. Therefore, open surgery for ESH could obtain good release of symptom for ESH.

However, there are many complications for open techniques, including large scar, wound complication, and slow recovery. Arthroscopic release of the ESH has become more common. Ilizaliturri et al. [22] reported that a total of 6 patients obtained complete resolution of symptoms after arthroscopic release of the iliopsoas tendon. Flanum et al. [23] showed that 6 patients with arthroscopic release at the lesser trochanter received 100% resolution of symptoms. El Bitar et al. [24], in their study with 55 patients, showed that 82% patients had excellent results. Although these studies showed good results in arthroscopic release for ESH, small sample size cannot be ignored as shortages for these articles. Therefore, we perform this study with over 200 patients to investigate the role of arthroscopic surgery for ESH. And the result of the current study was comparable to that of the surgical procedures in the previous studies.

Some limitations should be mentioned. First, inherent bias of retrospective analysis might be inevitable. Moreover, a lack of effective assessment for ESH resulted in difficulty in comparison with other studies. Finally, comparison between open and arthroscopic surgery was not performed in this study.

## Conclusions

In the present study, arthroscopic surgery could be an effective procedure for ESH, due to less operating time, small scar, fast postoperative recovery, and complete contracture release. These promising results of arthroscopic treatment of ESH need randomized trial to compare with open procedures.

## Abbreviations

BMI: Body mass index
ESH: External snapping hip
GM: Gluteus maximus
GT: Great trochanter
ITB: Iliotibial band
NSAID: Non-steroidal anti-inflammatory drugs
ROM: Range of motion
SHS: Snapping hip syndrome
